# Supplementary material for: A Consequence of Immature Breathing induces Persistent Changes in Hippocampal Synaptic Plasticity and Behavior: A Role of Pro-Oxidant State and NMDA Receptor Imbalance
Source: bioRxiv. 2023 Mar 21:2023.03.21.533692. Preprint. [Version 1] doi: 10.1101/2023.03.21.533692 (PMC10055328; doi:10.1101/2023.03.21.533692)
Supplement: Supplement 1 [file NIHPP2023.03.21.533692v1-supplement-1.pdf]

bioRxiv preprint doi: https://doi.org/10.1101/2023.03.21.533692; this version posted March 21, 2023. The copyright holder for this preprint (which was not certified by peer review) is the author/funder, who has granted bioRxiv a license to display the preprint in perpetuity. It is made available under aCC-BY 4.0 International license.

Postnatal Day	**Control** (N)	**nIH and nIH$_{Saline}$** (N)	**nIH$_{Mn}$** (N)	**nIH$_{REC\text{-}Mn}$** (N)	**P value**
4-5	2.59±0.06 (42)	2.48±0.08 (38)	2.43±0.08 (36)	2.52±0.10 (13)	P=0.50
14-15	6.17±0.12 (42)	6.24±0.27 (38)	5.88±0.14 (36)	6.98±0.019 (13)	P=0.29
55-60	28.32±0.78 (14)	29.49±0.66 (20)	28.78±0.60 (16)	30.11±0.66 (13)	P=0.43

Supplement 1: Control and nIH body mass at postnatal ages used.

bioRxiv preprint doi: https://doi.org/10.1101/2023.03.21.533692; this version posted March 21, 2023. The copyright holder for this preprint (which was not certified by peer review) is the author/funder, who has granted bioRxiv a license to display the preprint in perpetuity. It is made available under aCC-BY 4.0 International license.

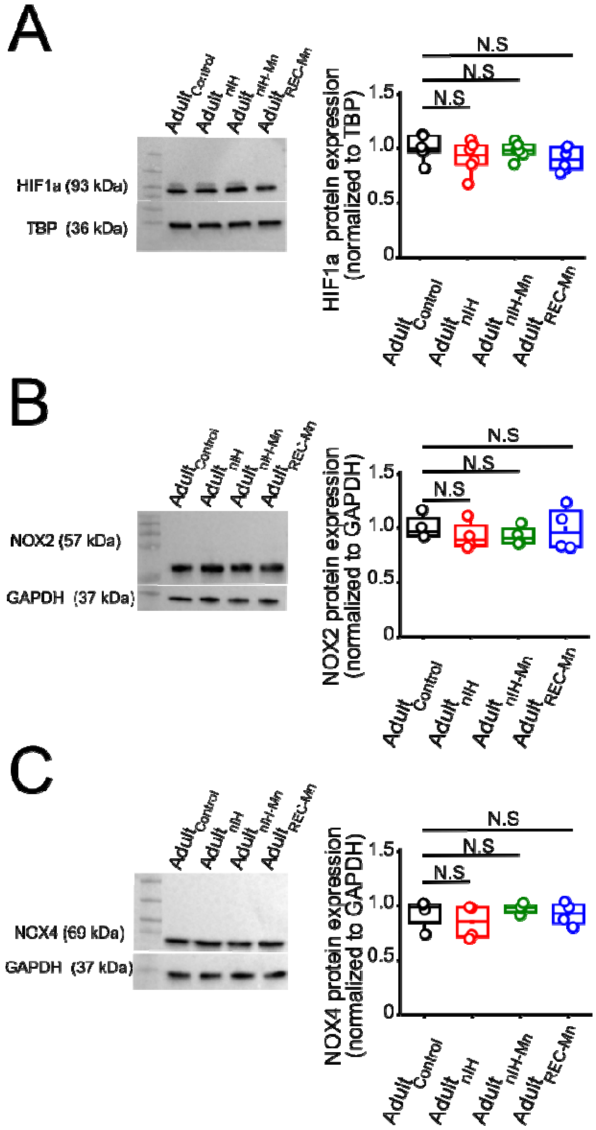

Supplement 2: Adult mice exposed to IH do not have increased HIF1a, NOX2 and NOX4 expression.

A. (*left*) Representative blot of nuclear HIF1a performed from adult mice unexposed ($Adult_{control}$), adult mice were exposed to nIH ($Adult_{nIH}$), adult mice were that received MnTMPyP during nIH exposure ($Adult_{nIH\text{-}Mn}$) and adult mice that receive MnTMPyP after nIH exposure ($Adult_{REC\text{-}Mn}$). (*right*). Quantification of nuclear HIF1a expression from adult control, $Adult_{nIH}$, $Adult_{nIH\text{-}Mn}$ and $Adult_{REC\text{-}Mn}$. (one way ANOVA, $F_{(3,20)}$=1.14; P=0.35, N=6). **B.** *(left)* Immunoblot of NOX2. (*right*) No significant differences was found in hippocampal homogenate from adult control, $Adult_{nIH}$, $Adult_{nIH\text{-}Mn}$ and $Adult_{REC\text{-}Mn}$ (one way ANOVA, $F_{(3,12)}$=10.35; P=0.78, N=4). **C.** (*left*) Representative image of NOX4. (*right*) Comparison of NOX4 expression between adult control, $Adult_{nIH}$, $Adult_{nIH\text{-}Mn}$ and $Adult_{REC\text{-}Mn}$. (one way ANOVA, $F_{(3,12)}$=0.79; P=0.51,N=4). The box plot parameters indicate mean ± S.E. The analysis was performed for A-C using one-way ANOVA followed by Bonferroni post hoc. N.S= no significant.

bioRxiv preprint doi: https://doi.org/10.1101/2023.03.21.533692; this version posted March 21, 2023. The copyright holder for this preprint (which was not certified by peer review) is the author/funder, who has granted bioRxiv a license to display the preprint in perpetuity. It is made available under aCC-BY 4.0 International license.

	**Adult$_{control}$** (N=14)	**P value**	**Adult$_{nIH}$ /Adult$_{nIH-Saline}$** (N=20)	**P value**
Velocity (m/s)	Session 1:0.058 ±0.003<br>Session 2:0.053 ±0.005<br>Session 3:0.049 ±0.005	P=0.70	Session 1:0.06±0.006<br>Session 2:0.054±0.005<br>Session 3:0.055±0.005	P=0.82
Distance traveled (m)	Session 1:18.53 ±2.05<br>Session 2:8.68 ±1.20<br>Session 3: 4.8 ±1.48	P<0.001	Session 1:20.58±2.56<br>Session 2:12.27±1.53<br>Session 3:7.36±1.73	P=0.006

Supplement 3: Velocity and distance in Adult$_{control}$ and Adult$_{nIH}$/Adult$_{nIH-Saline}$ during Barnes maze training sessions.

bioRxiv preprint doi: https://doi.org/10.1101/2023.03.21.533692; this version posted March 21, 2023. The copyright holder for this preprint (which was not certified by peer review) is the author/funder, who has granted bioRxiv a license to display the preprint in perpetuity. It is made available under aCC-BY 4.0 International license.

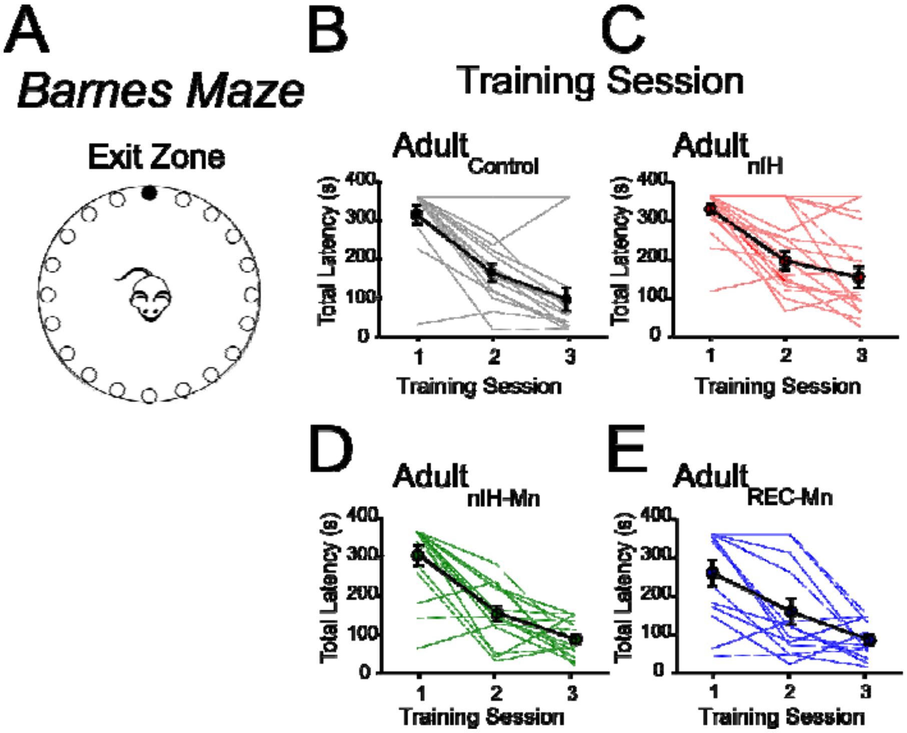

Supplement 4: Behavioral performance during training in the Barnes maze is evident across all experimental groups.

A. Barnes maze diagram. **B-C**. During the training session, the total latency average to the exit hole decreased over three training session in $\text{Adult}_{\text{control}}$ ($F_{(2,39)=18.47}$, P<0.0001, N=14) and $\text{Adult}_{\text{nIH}}$ ($F_{(2,57)=10.98)}$, P=0.0015, N=20). Black line represents average latency per trial whereas gray and red lines represent individual performance during training. **D-E.** Black lines represents average latency per trial whereas green and blues lines represent and individual latency during training (($\text{Adult}_{\text{nIH-Mn}}$) $F_{(2,45)=31.55}$, P<0.0001, N=16) and $\text{Adult}_{\text{REC-Mn}}$ ($F_{(2,36)=9.14}$, P=0.0006, N=13). The values indicate mean ± S.E. The analysis was performed for B-E using one-way ANOVA followed by Bonferroni post hoc.

bioRxiv preprint doi: https://doi.org/10.1101/2023.03.21.533692; this version posted March 21, 2023. The copyright holder for this preprint (which was not certified by peer review) is the author/funder, who has granted bioRxiv a license to display the preprint in perpetuity. It is made available under aCC-BY 4.0 International license.

	**$Adult_{control}$** (N=14)	**$Adult_{nIH}$/$Adult_{nIH\text{-}Saline}$** (N=20)	**P value**
Velocity (m/s)	0.05 ± 0.003	0.05 ±0.003	P=0.48
Distance traveled (m)	36.16±2	31.13 ±2.08	P=0.46
Time in the periphery (s)	361.3 ± 21.26	317.5± 23.16	P=0.17
Time in the center (s)	237.8±21.22	281.6 ±23.13	P=0.15

Supplement 5: Open field locomotor activity in $Adult_{control}$ and $Adult_{nIH}$/$Adult_{nIH\text{-}Saline}$.

bioRxiv preprint doi: https://doi.org/10.1101/2023.03.21.533692; this version posted March 21, 2023. The copyright holder for this preprint (which was not certified by peer review) is the author/funder, who has granted bioRxiv a license to display the preprint in perpetuity. It is made available under aCC-BY 4.0 International license.

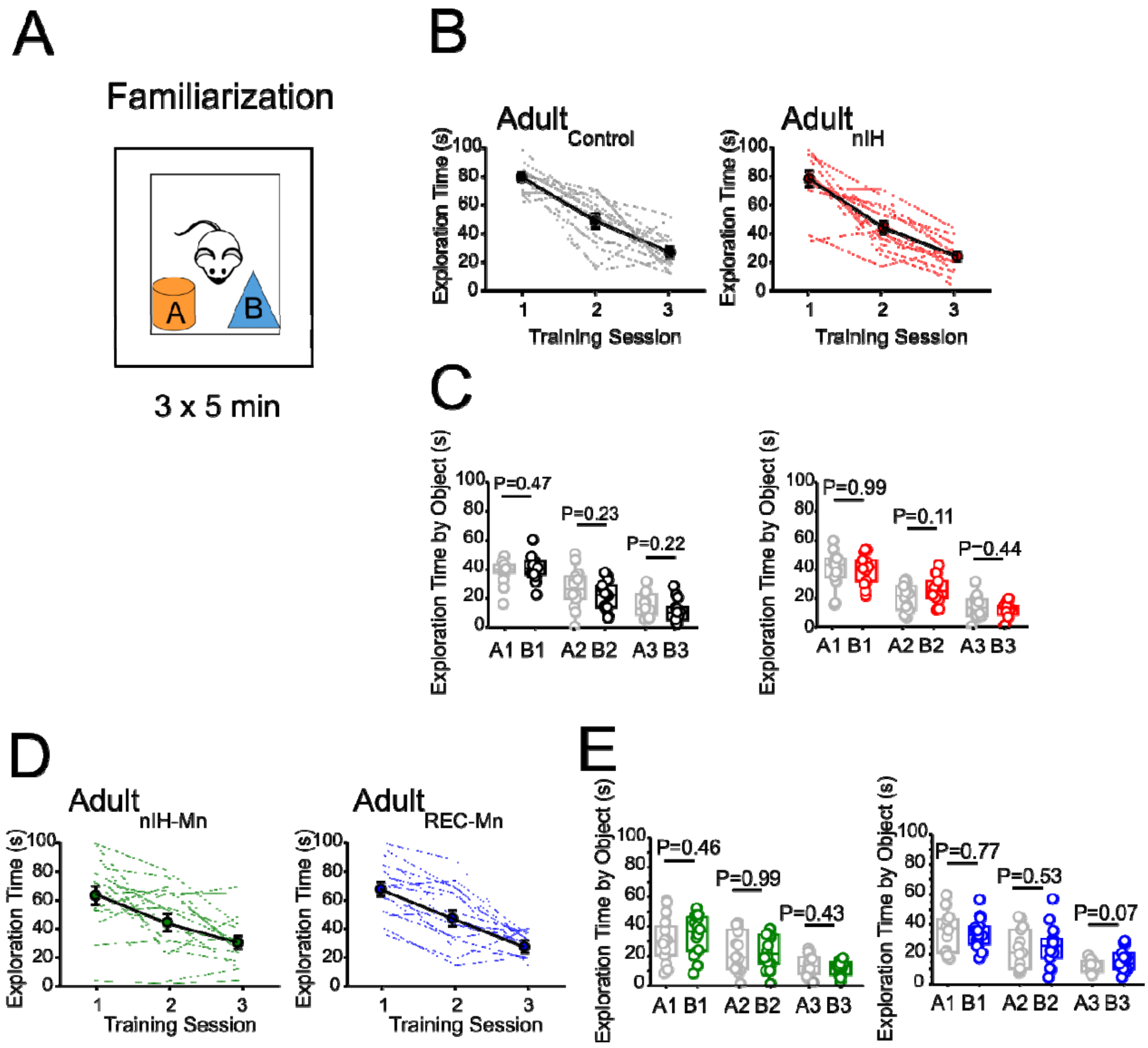

Supplement 6: Exploration of objects during Familiarization is similar in all experimental groups.

A. Object location diagram. **B.** In the familiarization phase, the exploration time in control ((left) $F_{(2,36)=39.05)}$, P<0.001; N=14) and IH ((right) $F_{(2,27)=18.33)}$, P<0.0001; N=20) decrease across to the three training session. Black line represents average latency per trial whereas grey and red represent individual performance during training. **C.** (left) Control mice have similar exploration time with the two objects in the first (t=0.80, df=23.94; P=0.47), second (t=1.52, df=22,63; P=0.23) and third (t =1.36, df=24; P=0.22) session. (right) IH mice explored the two-object similar time in the first session (t=0.06, df=17.08; P=0.99), second session (t=1.34, df=17.79; P=0.11) and third session (t=0.06, df=15.92; P=0.44). **D.** Mice received MnTMPyP during nIH exposure ($Adult_{nIH\text{-}Mn}$) and mice received MnTMPyP after nIH exposure ($Adult_{REC\text{-}Mn}$) decreased the exploration time across the three training session. $Adult_{nIH\text{-}Mn}$ ((left) $F_{(2,39)=6.10}$, P=0.0049, N=16) and $Adult_{REC\text{-}Mn}$ (right, $F_{(2,33)=14.33}$, P<0.0001, N=13). Black line represents average latency per trial whereas green and blue lines represent individual performance during training. **E.** (left) $Adult_{nIH\text{-}Mn}$ mice explored the two-object similar time in the first

bioRxiv preprint doi: https://doi.org/10.1101/2023.03.21.533692; this version posted March 21, 2023. The copyright holder for this preprint (which was not certified by peer review) is the author/funder, who has granted bioRxiv a license to display the preprint in perpetuity. It is made available under aCC-BY 4.0 International license.

(t=0.74, df=29.62; P=0.46), second (t=0.008, df=29.09; P=0.99) and third (t=0.78, df=26.35; P=0.43) sessions. (right) $Adult_{REC-Mn}$ mice spent similar time exploring the tow objects in the first (t=0.28, df=26.09; P=0.77), second (t=0.62, df=28.00; P=0.53) and third (t=1.88, df=20.67; P=0.07) sessions. The values indicate mean ± S.E. The analysis was performed for B and D using one-way ANOVA followed by Bonferroni post hoc. The analysis was performed for C and E using Paired- two-sided test.

bioRxiv preprint doi: https://doi.org/10.1101/2023.03.21.533692; this version posted March 21, 2023. The copyright holder for this preprint (which was not certified by peer review) is the author/funder, who has granted bioRxiv a license to display the preprint in perpetuity. It is made available under aCC-BY 4.0 International license.

	**Adult$_{nIH-Mn}$** (N=16)	**P value**	**Adult$_{REC-Mn}$** (N=13)	**P value**
Velocity (m/s)	Session 1:0.07 ±0.001<br>Session 2:0.07 ±0.003<br>Session 3:0.064 ±0.008	P=0.79	Session 1:0.07±0.02<br>Session 2:0.07±0.004<br>Session 3:0.065±0.012	P=0.82
Distance traveled (m)	Session 1:18.29 ±1.05<br>Session 2:10.42±1.28<br>Session 3: 5.32 ±0.82	P<0.001	Session 1:15.32±2.86<br>Session 2:7.84±1.31<br>Session 3:8.25±2.14	P=0.043

Supplement 7: Velocity and distance of MnTMPyP treated mice during Barnes maze training sessions.

bioRxiv preprint doi: https://doi.org/10.1101/2023.03.21.533692; this version posted March 21, 2023. The copyright holder for this preprint (which was not certified by peer review) is the author/funder, who has granted bioRxiv a license to display the preprint in perpetuity. It is made available under aCC-BY 4.0 International license.

	**Adult$_{nIH-Mn}$** (N=16)	**Adult$_{REC-Mn}$** (N=13)	**P value**
Velocity (m/s)	0.05 ± 0.003	0.055 ±0.002	P=0.33
Distance traveled (m)	35.21±1.45	33.25±1.24	P=0.35
Time in the periphery (s)	320.7 ± 26.31	304.1± 27.19	P=0.66
Time in the center (s)	272±24.65	280.2 ±27.12	P=0.82

Supplement 8 Open field locomotor activity in MnTMPyP treated subjects.
